# A Genome-Wide Association Study Reveals the Genetic Mechanisms of Nutrient Accumulation in Spinach

**DOI:** 10.3390/genes15020172

**Published:** 2024-01-28

**Authors:** Ni Ji, Zhiyuan Liu, Hongbing She, Zhaosheng Xu, Helong Zhang, Zhengwu Fang, Wei Qian

**Affiliations:** 1 MARA Key Laboratory of Sustainable Crop Production in the Middle Reaches of the Yangtze River (Co-Construction by Ministry and Province), College of Agriculture, Yangtze University, Jingzhou 434025, China; 2 State Key Laboratory of Vegetable Biobreeding, Institute of Vegetables and Flowers, Chinese Academy of Agricultural Sciences, Beijing 100081, China; 3 Zhongyuan Research Center, Chinese Academy of Agricultural Sciences, Xinxiang 453000, China

**Keywords:** spinach, nutrients, genome-wide association study, single-nucleotide polymorphism, candidate genes

## Abstract

Spinach is a significant source of vitamins, minerals, and antioxidants. These nutrients make it delicious and beneficial for human health. However, the genetic mechanism underlying the accumulation of nutrients in spinach remains unclear. In this study, we analyzed the content of chlorophyll a, chlorophyll b, oxalate, nitrate, crude fiber, soluble sugars, manganese, copper, and iron in 62 different spinach accessions. Additionally, 3,356,182 high-quality, single-nucleotide polymorphisms were found using resequencing and used in a genome-wide association study. A total of 2077 loci were discovered that significantly correlated with the concentrations of the nutritional elements. Data mining identified key genes in these intervals for four traits: chlorophyll, oxalate, soluble sugar, and Fe. Our study provides insights into the genetic architecture of nutrient variation and facilitates spinach breeding for good nutrition.

## 1. Introduction

The Spinach (*Spinacia oleracea* L.), belonging to the Amaranthaceae family, is primarily valued for its leaves as the main edible organ [1]. It is considered one of the healthiest vegetables in the human diet due to its richness in nutrients and health-promoting compounds, such as chlorophyll, vitamins, soluble sugars, and mineral elements, although it also contains some anti-nutritional factors, such as oxalate and nitrate [2]. The Food and Agriculture Organization of the United Nations (FAO) reports that in 2021, Asia produced 30.86 million tons of spinach, or 95.55% of the world’s total, with China producing 96.69% of that amount (https://www.fao.org/faostat (accessed on 25 October 2023)).

Spinach is a good source of nutrients, which is why it is popular among consumers. Mineral nutrients, for example, are required for plant and animal growth and development, and humans can only receive them via plant-based meals [3]. Among these elements, trace elements such as Fe, Mn, and Cu are important cofactors in the structure of certain enzymes and are essential in many biochemical pathways [4]. Crude fiber is a kind of dietary fiber made mostly of cellulose, hemicellulose, and lignin. It improves fecal volume, promotes intestinal peristalsis, prevents constipation and rectal cancer, and reduces obesity [5]. Additionally, cellulose, as a primary component of plant cell walls, directly influences the texture, palatability, and degree of tenderness of vegetables [6]. In addition to being rich in nutrients, spinach also contains more oxalate than most other crops [7]. Oxalate has been shown to exist in two forms: soluble oxalate and insoluble oxalate, with soluble oxalate being the predominant type found in spinach [8]. When absorbed by the digestive tract, oxalate is easily chelated by calcium ions, magnesium ions, and other metal ions to form insoluble oxalates, thus negatively affecting human health by reducing mineral absorption and promoting the formation of kidney stones [9,10].

Previous studies have discovered large variances in nutritional content within spinach; the reasons for these variations may include factors such as variety, growth conditions, and the environment [11,12,13,14]. In recent years, many studies have been conducted to evaluate spinach’s nutritional content and molecular basis. Fourteen mineral elements (Fe, Cu, Mn, and more) were examined in a panel of 292 spinach germplasm resources. Mineral element levels varied significantly, ranging from 50.43 to 138.58 μg/g for Fe, 2.22 to 10.97 μg/g for Cu, and 29.78 to 415.76 μg/g for Mn [14]. In one investigation, oxalate levels varied from 647.2 to 1056.2 mg/100 g (fresh weight) or from 53.4 to 79.9 mg/100 g (dry weight) [15]. Various methodologies have been used to identify the governing genes and relevant loci that influence these variances. Fourteen loci linked with Mn, Fe, and Cu contents in spinach were detected using a genome-wide association study (GWAS) with sequencing genotyping (GBS) data [14]. The GWAS study identified a potential area for oxalate at 915,444–1,022,853 bp on chromosome 5 [16]. Due to a scarcity of markers and the lack of a high-quality reference genome, potential genes related to these mineral elements are difficult to uncover.

GWAS is the study of phenotype–genotype relationships at the population level. It does not require the construction of genetic linkage maps; it can detect the genetic structure of phenotypes and identify numerous candidate genes [17]. When compared to approaches such as quantitative trait loci (QTL) mapping, it is faster, less expensive, and provides more precise localization. GWAS has been widely utilized to identify genes associated with quantitative traits in various crops, such as rice [18], maize [19], snap bean [20], tomato [21], cucumber [22], pepper [23], melon [24], and carrot [25]. For spinach, GWAS has been used to identify markers and genes associated with features such as disease resistance [26,27,28,29,30], sex determination [31], oxalate concentration [15], and mineral element content [14]. However, few GWASs have been reported on the comprehensive nutritional quality of spinach. In this study, the concentrations of chlorophyll a, chlorophyll b, oxalate, nitrate, crude fiber, soluble sugar, manganese, copper, and iron were determined in 62 inbred lines of spinach. These spinach lines were sequenced with around 10-fold genomic coverage. Using the resulting data, the GWAS for nine nutritional traits identified a substantial number of loci, including key genes regulating biosynthesis and the transportation of mineral elements, indicating that the GWAS of spinach could provide an effective approach for gene identification, pathway elucidation, and spinach improvement.

## 2. Materials and Methods

### 2.1. Plant Materials

A total of 62 spinach inbred lines were used in this experiment which were obtained from the Institute of Vegetables and Flowers at the Chinese Academy of Agriculture Sciences (CAAS). All accessions were planted in an open field in Beijing (40° N, 116° E) in the spring of 2019, with the same management practices as conventional cultivation. Young leaves were collected 25 days after sowing for resequencing. For nutrient content determination, 10–20 plants with uniform growth were selected from each accession 50 days after sowing.

### 2.2. Extraction and Determination of Nutrients

The extraction method was performed using the same protocol as before [32]. The chlorophyll content was determined by spectrophotometry and the organic acid content was determined using ion chromatography. Acid-base treatment was used to measure the crude fiber content. The soluble sugar content was determined using the copper reduction iodometric method. The concentration of mineral elements was determined by inductively coupled plasma emission spectrometry.

### 2.3. Statistical Analysis of Nutrient Content

IBM SPSS Statistics 23 software was used to calculate the mean value, standard deviation, and coefficient of variation of each component. Origin Pro 2022 software was used to determine the frequency distribution and correlation analysis and to generate figures.

### 2.4. DNA Extraction

Genomic DNA was extracted from the spinach leaves using the cetyltrimethylammonium bromide (CTAB) method [33]. The quality and concentration of DNA were detected using 1.0% agarose gel electrophoresis and an ND-1000 spectrophotometer (Thermo Fisher Scientific, Wilmington, NC, USA), respectively. The DNA solution was diluted to 20–100 ng/µL as a working solution and stored at −20 °C for subsequent testing.

### 2.5. Resequencing and Genotyping

After determining the extraction quality, the DNA was sent to the Berry Genomics Company (Beijing, China) for resequencing using the Illumina platform. The raw data were filtered using fastp software (v0.20.0) (parameter q 30, the rest were default) [34] to obtain the clean data. The clean reads were then aligned to the spinach reference genome Monoe-Viroflay [35] using Burrows–Wheeler Aligner (BWA) software (v0.7.17-r1188) (http://bio-bwa.sourceforge.net/ (accessed on 14 July 2023)). SAMtools software (v1.6-3-g200708f) [36] was used to sort the data, mark the duplicate sequences, and convert the alignment file into a binary alignment map (BAM) file. Single-nucleotide polymorphism (SNP) calling was performed using the genome analysis toolkit software (GATK4.3.0.0) [37]. Finally, raw SNPs were filtered using VCFtools software (v0.1.15) [38] based on the following criteria: deletion call rate > 0.8, minor allele frequency (MAF) < 0.05, loci with only two alleles to be retained, and information in all INFO fields to be retained without filtering. The high-quality SNPs obtained through these filtering criteria were used for GWAS analysis.

The functional annotation of variants was performed using ANNOVAR software (version: 2017-07-17) [39].

### 2.6. Linkage Disequilibrium Analysis

PopLDdecay software (v3.41) [40] was used to determine the linkage disequilibrium (LD) coefficient (r^2^) values for each pair of SNPs, perform genome-wide LD analysis, and visualize the results. LDBlockShow software (v1.40) [41] was utilized to identify LD blocks within the candidate regions.

### 2.7. Genome-Wide Association Analysis

The GWAS was performed using two statistical models in rMVP (https://github.com/xiaolei-lab/rMVP (accessed on 20 July 2023)): Generalized Linear Model (GLM) and Fixed and Random Model Circulating Probability Unification (FarmCPU) [42]. The kinship matrix (K) was calculated using the default method. Significance thresholds (−log_10_P) were corrected using Bonferroni correction. In this study, −log_10_P = 6.52 (P = 1/3356182) was used as the threshold value to determine the significance of SNP markers for loci associated with nutrient concentrations. The Manhattan plot was plotted using rMVp to visualize the GWAS analysis.

### 2.8. Candidate Gene Identification

Using Monoe-Viroflay as the reference genome, genes within the candidate region were extracted. The Blast Zone of TBtools II (https://github.com/CJ-Chen/TBtools-II/releases (accessed on 24 September 2023)) [43] was used to identify the corresponding homologous genes in *Arabidopsis thaliana*, and the one with the best score was taken as the homologous gene. The functions of the candidate genes were analyzed by combining the annotation information of the spinach genome and *A. thaliana* homologous genes.

## 3. Results

### 3.1. Statistical Analysis of Phenotyping Results

**Figure 1:**
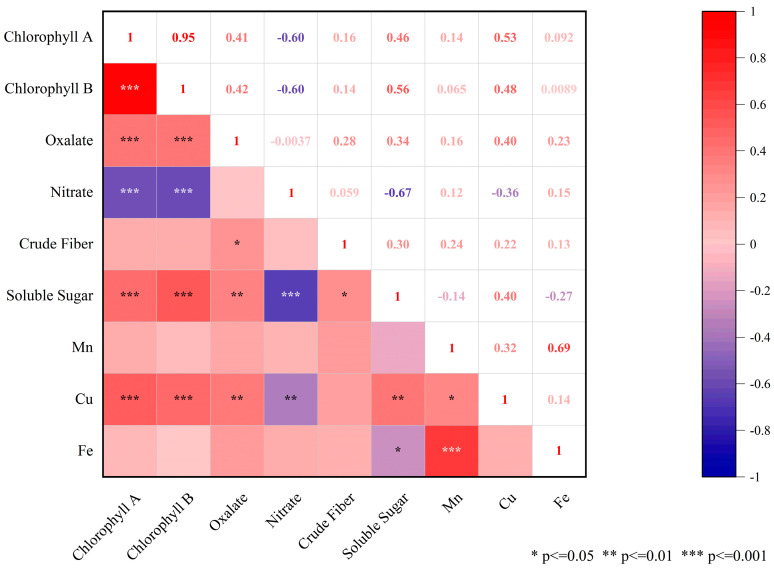
Correlation analysis of different nutritional elements in spinach.

In this study, the content of nine nutrients was investigated in different spinach accessions to reveal the change rule of the nutritional quality of spinach (Table 1 and Table S1). In terms of chlorophyll, the results showed that chlorophyll a and chlorophyll b in 62 spinach accessions were significantly correlated, and the correlation coefficient was as high as 0.95 (Figure 1). Specifically, the average chlorophyll a content was 0.749 mg/g, with a variance of 0.480–1.187 mg/g and a coefficient of variation of 23.541%. The average content of chlorophyll b was 0.308 mg/g, with values ranging from 0.170 to 0.466 mg/g, and the coefficient of variation was 26.739%. There was a significant variation in oxalate content among the test accessions. The average concentration was 4.614 g/kg, with a range of variation from 1.86 to 8.89 g/kg and a coefficient of variation of 29.509%. For nitrate, the average content was 0.996 g/kg, with a variance of 0.26 to 2.24 g/kg, exhibiting a relatively high coefficient of variation at 57.716%. Additionally, nitrate showed a significant negative correlation with chlorophyll a, chlorophyll b, and soluble sugars (Figure 1). For soluble sugars, the average content was 0.647%, with a variation range of 0.14–1.64%, and the coefficient of variation was 64.849%. For crude fiber, the average content was 0.728%, and it had the lowest coefficient of variation among all qualities at 8.714%. For mineral elements, the Fe content in spinach was much higher than that of Mn and Cu. The average Fe content was 135.524 mg/kg, and the average content of Mn and Cu was 6.560 and 1.235 mg/kg, respectively. Moreover, Fe and Mn showed a significant positive correlation, and the correlation coefficient was 0.69 (Figure 1). The content of all nine nutrients showed a normal distribution or an approximately normal distribution (Figure S1), aligning with the quantitative trait characteristics. This indicates that the content of these qualities is influenced or controlled by multiple genes.

### 3.2. Resequencing of Spinach Varieties

**Figure 2:**
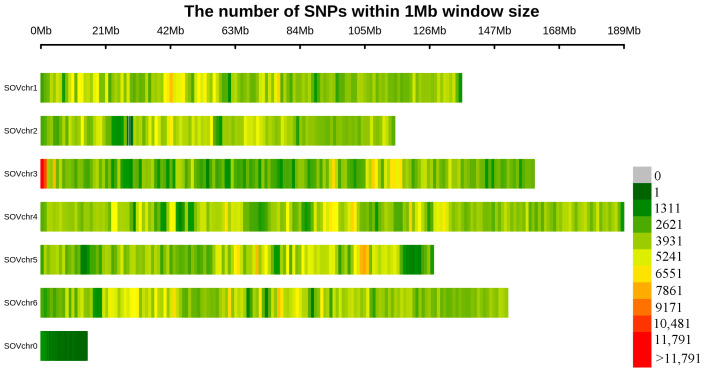
Distribution of 3,356,182 single-nucleotide polymorphisms (SNPs) among the six chromosomes of spinach within 1 Mb.

Resequencing was conducted on 62 spinach samples, resulting in a total of 787.7 GB of raw data. After filtering with the fastp software, 696.4 GB of clean data was retained. In alignment with the reference genome, Monoe-Viroflay identified a total of 7,728,915 SNPs. Following that, further filtering was performed to obtain 3,356,182 high-quality SNPs. Among these variations, 3,347,533 SNPs were anchored to chromosomes, with the majority located on chromosome 4 (734,008), while chromosome 2 had the fewest distributed SNPs (439,501) (Figure 2 and Table S2).

### 3.3. Genome-Wide Linkage Disequilibrium Analysis

**Figure 3:**
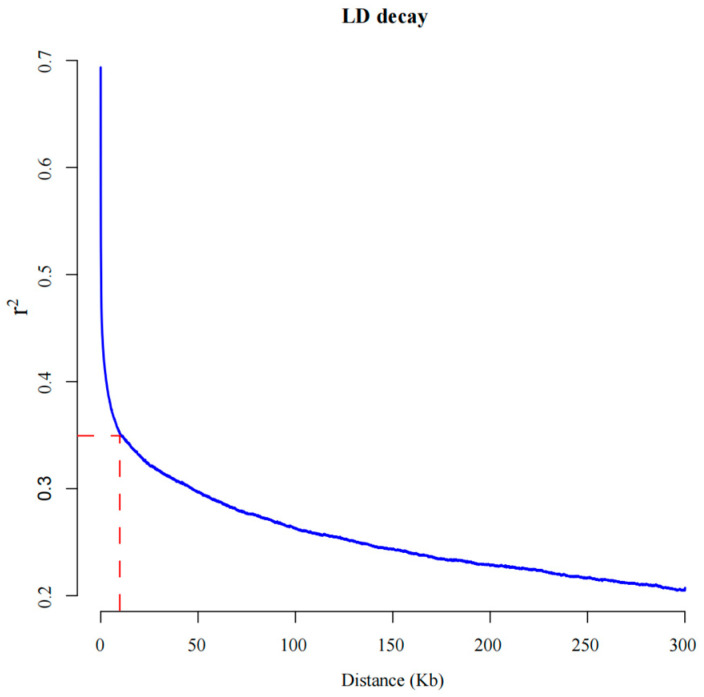
Linkage disequilibrium (LD) decay in the genome-wide association study (GWAS) population. The red dotted lines indicate the attenuation distance when the r-squared value of 0.35.

LD refers to the non-random association of alleles at different loci [44], reflecting the population’s recombination strength. It determines the marker density required and the accuracy of association analysis. During the genome-wide LD decay analysis of high-quality SNPs, when the r^2^ value decreased from its maximum of 0.7 to half of the maximum value (0.35), the corresponding physical distance was 10.47 kb (Figure 3). Therefore, the LD decay value for the entire spinach genome was 10.47 kb, which was faster than that in another study (5 kb) [45].

### 3.4. Genome-Wide Association Analysis of Nutrients

**Figure 4:**
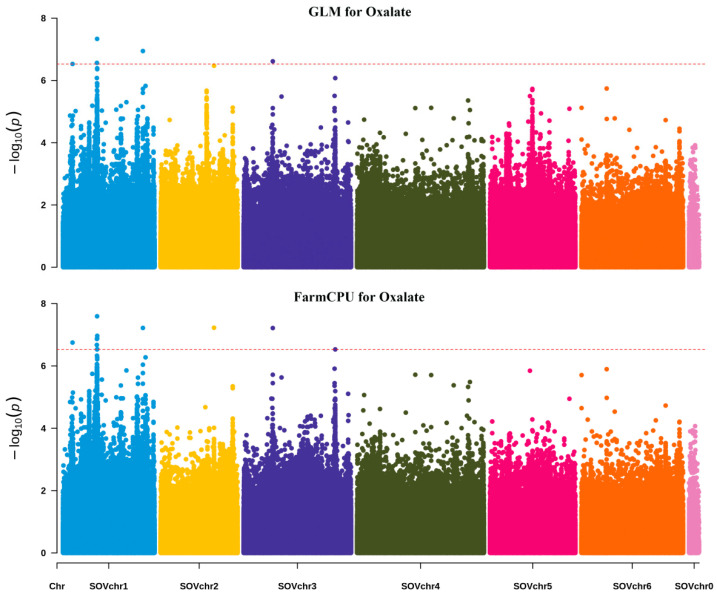
Manhattan plot for genome-wide association study (GWAS) of oxalate content in spinach using GLM and FarmCPU models. The horizontal and vertical axes represent the genomic position of the SNP and the association power for each SNP, respectively, with the trait expressed as −log_10_(p). The dashed line shows the Bonferroni-corrected genome-wide threshold.

Intending to identify the genetic loci controlling these traits, we utilized 3,356,182 SNPs based on two models (GLM and FarmCPU) in rMVP to conduct a GWAS for nine nutrient concentrations in spinach. The GWAS significance threshold was determined to be −log_10_(p) = 6.52 using the Bonferroni correction. Based on GLM and FarmCPU, we found 1996 and 104 linked loci, respectively, with 23 loci recognized by both models (Table 2 and Table S3; Figure 4, Figures S2 and S3). All in all, there were 2077 loci with −log_10_(p) > 6.52, mainly distributed on chromosomes 1, 2, and 4. As for chlorophyll a and chlorophyll b, the peak signal was located at the same locus, SOVchr4-65150089; in addition, they co-localized on chromosomes 1, 4, and 6. For oxalate, nitrate, and Fe, their lead SNPs were distributed at 50,807,332, 64,960,432, and 43,602,473 bp on chromosome 1, respectively. Strong association signals were located on chromosome 2 for soluble sugar and Cu and on chromosome 5 for Mn. However, no associated loci were identified for crude fiber.

### 3.5. Candidate Genes

To find candidate genes, we conducted a haplotype analysis of SNPs throughout the whole genome using LDBlockShow. The region associated with the significant locus was identified as a candidate interval. Subsequently, we screened candidate genes related to the target traits based on the annotation information of the spinach genome Monoe-Viroflay. Among the candidate regions, we identified 127 genes, including 19 unknown genes and 108 known annotation genes, of which 85 had homologous genes in *A. thaliana* (Table S4).

Regarding chlorophyll, within its candidate regions, we identified a total of 62 predicted genes. Among them, 50 genes were connected with chlorophyll a content, 8 with chlorophyll b, and 4 with both. Among the 62 genes, based on gene annotation, 4 genes were discovered to be associated with chlorophyll content. *SOV1g020220* was located on chromosome 1: 93,828,109–93,830,147 bp, and was associated with both chlorophyll a and chlorophyll b. Within its coding region, we identified four non-synonymous SNPs, leading to amino acid changes (Table S5). Additionally, it served as a homologous gene to *ATFRD3*, which encoded a multidrug and toxin efflux (MATE) family member and played a critical role in regulating cellular iron ion balance; another study suggested that iron plays a role in the biosynthesis of chlorophyll [4]. *SOV4g012830* was found in the candidate region of chlorophyll a, was homologous to *ATFYH3* in *A. thaliana*, and was a component of the PHYTOCHROME A (PHYA) signaling network, mediating the Far-Red High-Irradiance Response (FR-HIR) in collaboration with FAR-RED IMPAIRED RESPONSE1 (FAR1). *SOV4g012890* encoded a FAR1 domain-containing protein, and two non-synonymous SNPs were identified in its coding region (Table S5). The last gene was *SOV4g004990,* correlated with chlorophyll b concentration, and its homologous gene *AT2G01050* regulated zinc ion binding in *A. thaliana*. Similarly, zinc ions have been demonstrated to participate in the synthesis of chlorophyll [46].

For oxalate, we found a total of 12 genes associated with its content, among which *SOV1g009820* was considered a key candidate gene. *SOV1g009820,* which encodes β-glucuronosyltransferase GlcAT14A, is found on SOVchr1 at 50,879,476–50,883,262 bp. Furthermore, its coding region exhibited extensive variation, including 11 non-synonymous SNPs, 3 synonymous SNPs, 1 stop-gain SNP, two frameshift insertions, and one non-frameshift deletion. This stop-gain caused the gene to terminate prematurely, resulting in a protein with just 215 amino acids (Table S5).

For soluble sugar, within the range of SOVchr2: 108,223,175–108,323,175 bp, we identified eight genes. Among them, *SOV2g035100* was located 12 kb upstream of the SOVchr2-108273175 locus and was thought to be an essential gene that regulates soluble sugar concentration. *SOV2g035100* was homologous to *ATSTP10*, which encoded a hexose-H (+) symporter in *A. thaliana*.

With regard to Fe, we discovered three genes in their LD blocks, two of which were located on chromosome 1 and one was located on chromosome 5. *SOV1g008480* was considered to be a potential gene affecting iron content in spinach. It was located within the range of SOVchr1: 43,699,103–43,701,699 bp and was homologous to *ATARD1*. It encodes an acireductone dioxygenase that is activated by contact with a heterotrimeric G protein β subunit, acting as a metal enzyme that may regulate iron ion binding.

## 4. Discussion

With the improvement of people’s living standards, growing attention has gradually turned to health and nutrition. Spinach, as a representation of comprehensive nutrition, has progressively gained recognition. As a result, researchers are concerned not only with the yield of spinach but also with increasing the concentrations of beneficial nutrients while decreasing the concentrations of the harmful components of spinach. To better understand the genetic mechanism, several efforts have been made to assess dietary features using various methodologies [32].

In order to identify genes regulating nutrient components in spinach, our study used individual nutrient content and resequencing data, conducting a GWAS analysis of nine nutritional elements of 62 spinach accessions. GWAS has been employed for a variety of crops to uncover linked loci or genes for nutritional elements such as soluble sugars [47] and mineral elements [48]. This shows that GWAS is appropriate for studying nutritional elements. Moreover, past studies have shown that GWAS is not only successful in varied sample investigations but also produces important results, even in studies with less than 100 samples [49]. As a consequence, the number of accessions has no bearing on the credibility of this study’s findings. Spinach is a hybrid species with a high effective recombination rate, which results in a substantial number of heterozygotes and a low LD. Our calculated LD decay distance for the whole spinach genome was 10.47 Kb, with an r-squared value of 0.35. This decay distance is remarkably shorter than that of other crops, such as snap bean [50] and rice [51]. Rapid LD decay in spinach is advantageous for determining the correlation between linked loci, thereby reducing the risk of false positives in candidate gene identification during GWAS.

Chlorophyll is a natural pigment found in plants that plays an important role in the photosynthesis process. It has a variety of activities, including anticancer, antibacterial, and antioxidant properties. Additionally, chlorophyll plays a protective role in photosynthesis by shielding plants from the damage caused by light during complex cellular processes [52]. In plants, chlorophyll a can synthesize chlorophyll b, which may then be transformed back into chlorophyll a [53]. In previous studies, a gene in Chlamydomonas called *CAO* (chlorophyll a oxygenase) has been discovered that codes for an enzyme that oxidizes chlorophyll a to produce chlorophyll b [54]. This study discovered four chlorophyll-related candidate genes. Among them, *SpFRD3* (*SOV1g020220*) is a homolog of *ATFRD3*, which is implicated in iron ion transport and balance in *A. thaliana* [55]. Previous research has indicated that iron, as a redox-active metal, is involved in photosynthesis and the formation of chlorophyll in plants [4]. Another potential gene, *SOV4g004990*, is homologous to *AT2G01050* and is involved in binding zinc ions. Zinc ions, which act as cofactors, are essential components of numerous enzymes and participate in chlorophyll synthesis [46]. Therefore, we suggest that *SpFRD3* and *SOV4g004990* may influence the chlorophyll content in spinach by regulating the transport or binding of metal ions within the plant. *SpFHY3* (*SOV4g012830*) is homologous to *ATFHY3* (*AT3G22170*), which is a component of the PHYA signaling network, mediating the FR-HIR response to far-red light in conjunction with FAR1. In *A. thaliana*, *ATFHY3* positively regulates chlorophyll biosynthesis by activating the expression of the *HEMB1* gene (*HEMB1* encodes 5-aminolevulinic acid dehydratase in the chlorophyll biosynthetic pathway) [56]. Additionally, *SOV4g012890* encodes a FAR1 domain-containing protein. The Far1 nuclear protein is derived from the plant MuDR transposase, involved in phytochrome signaling [57], and it has been reported that photosensitive pigments enter the nucleus of the cell when exposed to light and interact directly with transcription factors to regulate gene expression and indirectly affect chlorophyll biosynthesis [58,59]. Therefore, we hypothesize that *SpFHY3* and *SOV4g012890* may influence the chlorophyll content in spinach by regulating the transmission of photosensitive pigment signals within the plant. In future research, we will use virus-induced gene silencing (VIGS) to confirm the functions of these two genes.

In spinach, the occurrence of a substantial amount of oxalate has an influence on both taste and health. The distribution of oxalate is affected by a variety of factors, including the environment, season, growing period, and genetics. In spinach, researchers have observed higher oxalate concentrations in older leaves [60]; the oxalate content in spinach petioles is lower than that in leaves, thus recommending the selection and breeding of cultivars with high leaf/petiole ratios [61]. Fall-planted spinach has the lowest oxalate content, followed by spring- and summer-planted spinach, while winter-planted spinach has the highest. [62]. Previous studies have identified two genes on chromosome 5 encoding metal ion transporter proteins that affect the levels of soluble oxalate in spinach by influencing the levels of calcium and other ions in spinach cells, thereby affecting the levels of soluble oxalate in spinach [16]. Despite the fact that spinach oxalate has been extensively studied by other researchers, the identified loci or genes have not been validated, and the results of previous studies are not consistence, indicating that oxalate is controlled by multilocus genes and is highly influenced by genetic background and environmental differences. The study identified 12 genes connected to oxalate, and *SOV1g009820* encodes β-glucuronosyltransferase, which transfers glucuronic acid to Arabinogalactan-proteins (AGPs). Additionally, glucuronic acid can bind to calcium ions [63]. Moreover, there is one stop-gain SNP in the coding region of this gene that contributes to the premature termination of the expression of this gene and may decrease the activity of glucuronosyltransferase, which, in turn, increases the binding of glucuronide and calcium ions in spinach and decreases the binding of oxalate and calcium ions, leading to a higher soluble oxalate content in spinach. Therefore, we consider this gene as an important candidate gene that is related to the oxalate content of spinach.

Soluble sugars play a crucial role in regulating the taste of fruits and vegetables [17]. They provide energy and structural material and are significant regulators of plant growth, development, and gene expression [64]. It has been demonstrated that soluble sugar synthesis and transporter genes are associated with their concentration accumulation in crops. For example, in Chinese cabbage, there are six genes associated with soluble sugar content, *BraA05gAOP1*, *BraA04gAOP4*, *BraA03gHT7*, and *BraA01gHT4* (hexose transporter protein), which are structural genes in soluble sugar biosynthesis, and *BraA01gCHR11* and *BraA07gSCL1*, which are two important transcription factors regulating soluble sugar biosynthesis [65]. There are few reports on loci or genes related to soluble sugar content in spinach. Among the four genes identified in this study, the *A. thaliana* homolog of *SpSTP10* (*SOV2g035100*), *ATSTP10,* encodes a hexose-H (+) symporter and catalyzes the high-affinity uptake of glucose, galactose, and mannose [66]. This is consistent with earlier studies that suggested that this gene may be involved in the biosynthesis of soluble sugars.

Iron plays an important role in both humans and plants. First, in the human body, iron is a component of hemoglobin, myoglobin, cytochromes, and some respiratory enzymes, and is involved in oxygen and carbon dioxide transport and exchange and tissue respiration in the body [67]. Second, for plants, iron is a catalyst for chlorophyll synthesis, participates in photosynthesis, and affects root development and overall growth [68]. This study found *SpARD1* (*SOV1g008480*) to be a putative iron-related gene. The homologous gene of this gene is *ATARD1* (*AT4G14716*), a metalloenzyme that helps *A. thaliana* bind iron ions and acireductone dioxygenase (ARD). Within the iron ion-related pathway in *A. thaliana*, *ATARD1* catalyzes the conversion of acid-reduced ketones to formate and 2-keto-4-methylthiobutyrate as part of the iron ion-related pathway [69]. Thus, based on findings from previous research, we hypothesize that *SpARD1* may play a similar role in spinach, participating in the binding of iron ions. We will apply the genetic transformation to the candidate gene in order to assess it more thoroughly.

Molecular marker-assisted selection (MAS) breeding makes use of the fact that molecular markers are closely linked to the genes controlling the target traits and selects excellent individuals with the target traits by detecting molecular markers in the progeny. Compared with traditional breeding methods, this method is faster, more precise, and less affected by environmental circumstances. It may be employed in a number of breeding processes, including identifying parentage, transmitting quantitative and recessive traits in backcrossing, selecting hybrid progeny, predicting hybrid advantage, and determining variety purity [70]. Except for crude fiber, this analysis discovered 2077 SNP loci connected with the remaining eight characteristics. Particularly for Fe, we have identified two associated loci, located at 43,602,473 bp on chromosome 1 and 91,913,409 bp on chromosome 5. These loci accounted for 42.03% and 48.63% of the phenotypic variance, respectively. Both SNPs demonstrated unusually high phenotypic variability and might be utilized to develop molecular markers to aid in the selection of Fe-rich spinach types. In conclusion, the SNP loci associated with nutrient elements identified in this study can be developed as molecular markers, and it is possible to polymerize these markers to screen for superior spinach varieties that are rich in chlorophyll, soluble sugars, and mineral elements, as well as containing small amounts of oxalic acid and nitrate in spinach germplasm.

## 5. Conclusions

This study identified 2077 SNP loci associated with the concentrations of chlorophyll a, chlorophyll b, oxalate, nitrate, soluble sugars, Fe, Mn, and Cu in spinach. We predicted seven candidate genes based on these loci. Among them, *SpFRD3*, *SOV4g004990*, *SpFHY3*, and *SOV4g012890* were considered to be associated with chlorophyll. They influence the chlorophyll content in spinach through the regulation of iron ion balance, zinc ion binding, and photosensitive pigment signal transduction. Additionally, *SOV1g009820* encodes β-glucuronosyltransferase, *SpSTP10* encodes a hexose-H (+) symporter, and *SpARD1* is involved in iron ion binding. These genes are associated with oxalate, soluble sugar, and iron content, respectively. Therefore, this study provides SNP locus information and potential candidate genes for enhancing spinach’s positive traits and diminishing its negative ones. These SNP loci should be used in subsequent research to provide molecular markers for molecular-assisted breeding. Additionally, further research is needed to explore these genes and gain a deeper understanding of their molecular mechanisms in altering the nutritional quality of spinach. Such investigations contribute to a better understanding of the regulatory mechanisms of plant nutritional quality, offering a scientific basis for optimizing spinach’s nutritional characteristics.

## Figures and Tables

**Table 1:** The average, range, standard deviation, and coefficient of variation of nine nutrients in spinach.

Trait	Average	Minimum	Maximum	Range	Standard Deviation	Coefficient of Variation (%)
Chlorophyll A (mg/g)	0.749	0.480	1.187	0.707	0.176	23.541
Chlorophyll B (mg/g)	0.308	0.170	0.466	0.296	0.082	26.739
Oxalate (g/kg)	4.614	1.860	8.890	7.03	1.360	29.509
Nitrate (g/kg)	0.996	0.260	2.240	1.98	0.575	57.716
Crude Fiber (%)	0.728	0.590	0.870	0.28	0.063	8.714
Soluble Sugar (%)	0.647	0.140	1.640	1.5	0.420	64.849
Mn (mg/kg)	6.56	4.00	10.70	6.7	1.369	20.864
Cu (mg/kg)	1.235	0.91	3.30	2.39	0.329	26.626
Fe (mg/kg)	135.542	77	265	188	32.201	23.757

**Table 2:** Information on SNPs associated with nutrient elements detected using the FarmCPU model.

Trait	SNP	Chr.	Position	Alleles		−Log10(P)	R-Square(%)
Chlorophyll A	SOVchr4-65150089	4	65150089	C	A	10.93	0.14
	SOVchr4-6134363	4	6134363	G	T	10.91	0.11
	SOVchr4-63008113	4	63008113	G	C	10.54	0.12
	SOVchr1-96376944	1	96376944	C	A	9.55	0.08
	SOVchr6-145880662	6	145880662	T	G	8.13	0.06
	SOVchr4-26424208	4	26424208	G	A	6.44	0.07
Chlorophyll B	SOVchr4-65150089	4	65150089	C	A	10.93	0.06
	SOVchr4-105925437	4	105925437	G	T	8.82	0.04
	SOVchr4-145620388	4	145620388	G	T	7.75	0.02
	SOVchr4-179951205	4	179951205	A	G	7.30	0.03
	SOVchr1-93842468	1	93842468	A	C	7.16	0.03
	SOVchr6-87982689	6	87982689	T	A	6.94	0.05
	SOVchr4-6134363	4	6134363	G	T	6.80	0.03
	SOVchr4-163832653	4	163832653	G	A	6.70	0.03
Oxalate	SOVchr1-50807332	1	50807332	G	T	7.59	2.59
	SOVchr2-79590343	2	79590343	T	C	7.23	1.32
	SOVchr1-119069592	1	119069592	T	C	7.22	1.17
	SOVchr3-43424516	3	43424516	C	T	7.22	2.41
	SOVchr1-50996180	1	50996180	G	T	6.97	1.23
	SOVchr1-50996185	1	50996185	T	C	6.97	1.23
	SOVchr1-50934532	1	50934532	C	T	6.88	1.25
	SOVchr1-50565605	1	50565605	C	T	6.87	1.44
	SOVchr1-14238191	1	14238191	A	G	6.75	1.49
	SOVchr1-50453692	1	50453692	C	T	6.67	1.20
	SOVchr1-50453768	1	50453768	G	A	6.67	1.20
	SOVchr1-51019015	1	51019015	T	C	6.54	1.22
	SOVchr1-51024100	1	51024100	A	C	6.54	1.22
	SOVchr1-50731214	1	50731214	C	T	6.53	1.23
	SOVchr3-136466183	3	136466183	T	C	6.53	1.76
Nitrate	SOVchr1-64960432	1	64960432	T	G	7.41	0.65
	SOVchr1-64744070	1	64744070	C	T	7.34	0.59
	SOVchr4-148946915	4	148946915	G	T	7.32	0.63
	SOVchr4-148947017	4	148947017	A	G	7.32	0.63
	SOVchr1-64639785	1	64639785	A	C	7.28	0.56
	SOVchr1-64726231	1	64726231	A	G	7.28	0.56
	SOVchr1-64873483	1	64873483	T	C	7.21	0.60
	SOVchr1-64971908	1	64971908	T	G	7.21	0.60
	SOVchr1-64636577	1	64636577	A	T	7.21	0.60
	SOVchr1-64255238	1	64255238	A	T	6.99	0.62
	SOVchr1-122781901	1	122781901	T	C	6.95	0.40
	SOVchr2-66337447	2	66337447	C	A	6.85	1.04
	SOVchr1-122779934	1	122779934	C	T	6.84	0.38
	SOVchr1-122779963	1	122779963	T	C	6.84	0.38
	SOVchr1-122779967	1	122779967	C	T	6.84	0.38
	SOVchr1-64826287	1	64826287	T	C	6.80	0.52
	SOVchr1-64490849	1	64490849	A	G	6.75	0.54
	SOVchr1-64740737	1	64740737	T	C	6.75	0.54
	SOVchr1-64781014	1	64781014	T	C	6.75	0.54
	SOVchr1-64983706	1	64983706	C	A	6.75	0.54
	SOVchr1-65008639	1	65008639	T	G	6.75	0.54
	SOVchr1-122781886	1	122781886	A	T	6.71	0.39
	SOVchr1-94072908	1	94072908	C	T	6.69	0.43
	SOVchr4-148944398	4	148944398	T	G	6.69	0.67
	SOVchr1-64667101	1	64667101	A	C	6.67	0.61
	SOVchr1-64675881	1	64675881	G	C	6.67	0.61
	SOVchr1-64686189	1	64686189	T	A	6.67	0.61
	SOVchr1-64772753	1	64772753	A	G	6.67	0.61
	SOVchr1-64772980	1	64772980	T	C	6.67	0.61
	SOVchr1-64782044	1	64782044	T	G	6.67	0.61
	SOVchr1-64782074	1	64782074	G	A	6.67	0.61
	SOVchr1-64858451	1	64858451	T	G	6.67	0.61
	SOVchr1-64859462	1	64859462	C	T	6.67	0.61
	SOVchr1-64871632	1	64871632	T	G	6.67	0.61
	SOVchr1-64879489	1	64879489	T	C	6.67	0.61
	SOVchr1-64894852	1	64894852	A	G	6.67	0.61
	SOVchr1-64898345	1	64898345	A	G	6.67	0.61
	SOVchr1-64899576	1	64899576	T	C	6.67	0.61
	SOVchr1-64902305	1	64902305	T	C	6.67	0.61
	SOVchr1-64905439	1	64905439	G	A	6.67	0.61
	SOVchr1-64916919	1	64916919	G	C	6.67	0.61
	SOVchr1-64924310	1	64924310	A	G	6.67	0.61
	SOVchr1-64949052	1	64949052	T	G	6.67	0.61
	SOVchr1-64972031	1	64972031	T	G	6.67	0.61
	SOVchr1-64993886	1	64993886	A	C	6.67	0.61
	SOVchr1-65009190	1	65009190	T	C	6.67	0.61
	SOVchr1-65011906	1	65011906	G	C	6.67	0.61
	SOVchr1-65439679	1	65439679	T	C	6.67	0.61
	SOVchr1-122780968	1	122780968	G	A	6.65	0.38
	SOVchr1-36836579	1	36836579	C	T	6.63	0.47
	SOVchr1-64730231	1	64730231	T	G	6.56	0.56
	SOVchr1-64820621	1	64820621	T	C	6.56	0.56
	SOVchr1-64906356	1	64906356	A	C	6.56	0.56
	SOVchr1-9323565	1	9323565	A	G	6.54	0.47
	SOVchr4-161857844	4	161857844	C	T	6.53	0.57
	SOVchr4-161857853	4	161857853	G	A	6.53	0.57
	SOVchr4-161857864	4	161857864	G	T	6.53	0.57
	SOVchr4-161857871	4	161857871	G	A	6.53	0.57
	SOVchr1-65248580	1	65248580	A	C	6.53	0.46
Soluble Sugar	SOVchr4-62896056	4	62896056	G	A	7.15	0.16
	SOVchr1-122779550	1	122779550	T	A	6.89	0.09
	SOVchr2-78543061	2	78543061	C	T	6.87	0.18
Mn	SOVchr5-42208975	5	42208975	A	G	7.06	1.27
	SOVchr5-40744482	5	40744482	G	A	6.80	1.67
	SOVchr4-54452300	4	54452300	C	T	6.58	1.92
Cu	SOVchr2-26477848	2	26477848	A	G	31.54	1.36
	SOVchr1-6225211	1	6225211	C	A	26.58	0.32
	SOVchr3-33311145	3	33311145	T	C	10.51	0.12
	SOVchr3-136997057	3	136997057	T	C	9.49	0.10
	SOVchr4-25830607	4	25830607	A	T	8.73	0.05
	SOVchr5-91037611	5	91037611	A	C	7.81	0.09
	SOVchr6-66852190	6	66852190	T	C	6.93	0.08
	SOVchr4-5213538	4	5213538	C	T	6.75	0.04
Fe	SOVchr1-43602473	1	43602473	C	A	7.14	42.40
	SOVchr5-91913409	5	91913409	T	C	6.71	48.32

## Data Availability

Data are contained within the article and Supplementary Materials.

## Supplementary Materials

The following supporting information can be downloaded at https://www.mdpi.com/article/10.3390/genes15020172/s1. Figure S1: Frequency distribution of different nutrient elements in 62 spinach accessions; Figure S2: Manhattan plot for genome-wide association analysis (GWAS) of nutrient (a: chlorophyll a; b: chlorophyll b; c: nitrate; d: soluble sugar; e: Mn; f: Cu; and g: Fe) content in spinach using GLM and FarmCPU models. The horizontal and vertical axes represent the genomic position of the SNP and the association power for each SNP, respectively, with the trait expressed as −log_10_(p). The dashed line shows the Bonferroni-corrected genome-wide threshold; Figure S3: Map of the distribution of SNPs associated with nutrient content on each chromosome. Table S1: Comparative analysis of nutritional quality of different spinach accessions; Table S2: SNP distribution on chromosomes; Table S3: Information on SNPs associated with the nutrient elements detected using the GLM model; Table S4: Putative genes associated with the nutritional composition of spinach within candidate regions based on associated SNPs; Table S5: Functional annotation information for exonic regions of candidate genes.
