# Supplementary material for: A Genome-Wide Association Study Reveals the Genetic Mechanisms of Nutrient Accumulation in Spinach
Source: Genes (Basel). 2024 Jan 28;15(2):172. doi: 10.3390/genes15020172 (PMC10887921; doi:10.3390/genes15020172)
Supplement: Supplementary file 1 [file genes-15-00172-s001.zip › Figure S2.pdf]

a

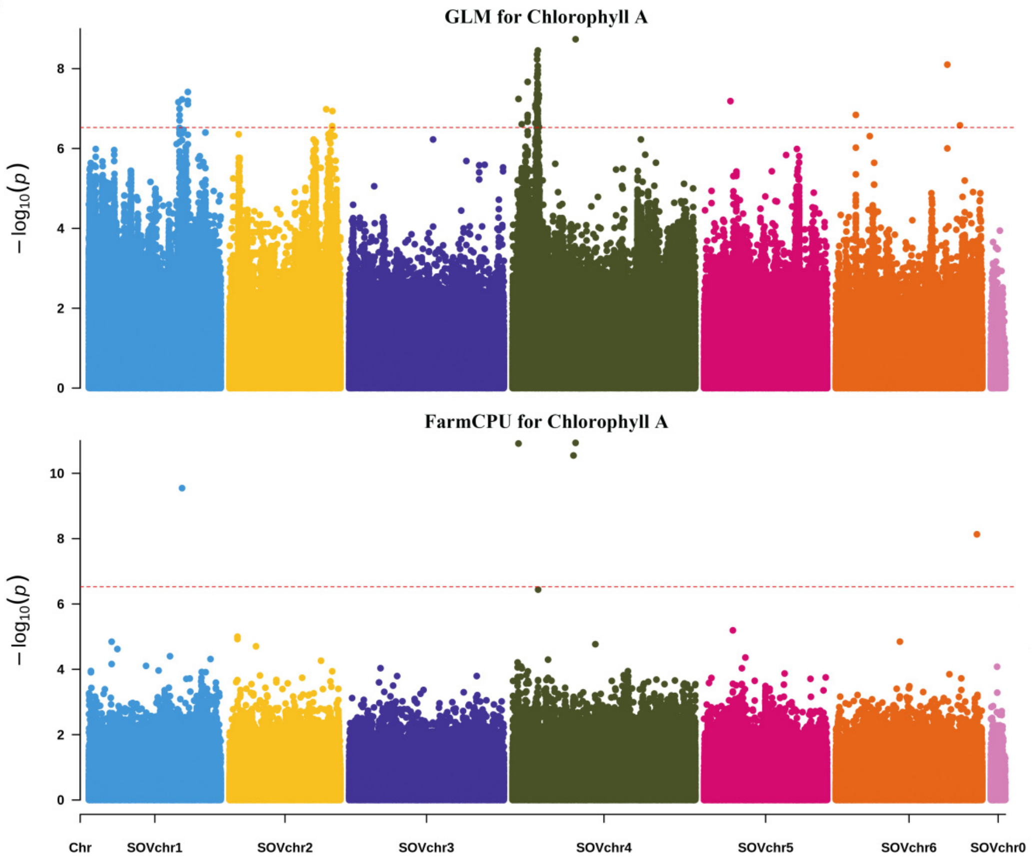

GLM for Chlorophyll A
FarmCPU for Chlorophyll A
$-\log_{10}(p)$
$-\log_{10}(p)$
0
2
4
6
8
10
Chr
SOVchr1
SOVchr2
SOVchr3
SOVchr4
SOVchr5
SOVchr6
SOVchr0

b

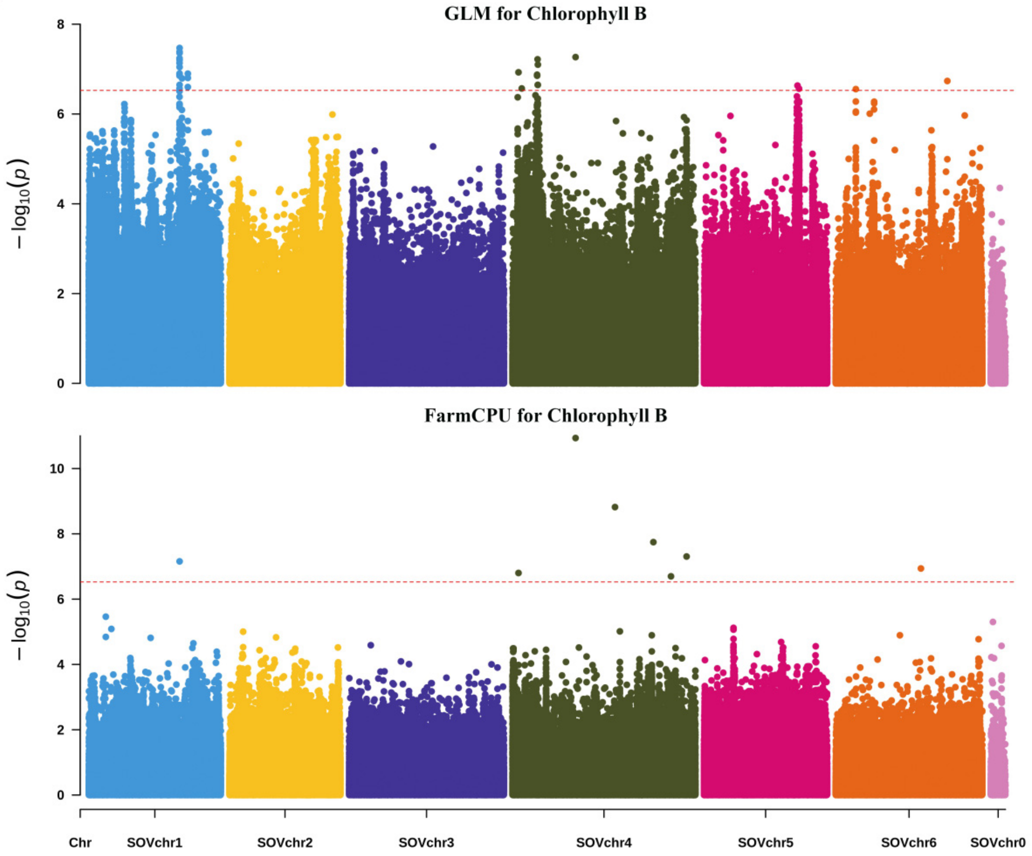
GLM for Chlorophyll B
FarmCPU for Chlorophyll B
$-\log_{10}(p)$
$-\log_{10}(p)$
0
2
4
6
8
10
Chr
SOVchr1
SOVchr2
SOVchr3
SOVchr4
SOVchr5
SOVchr6
SOVchr0

c

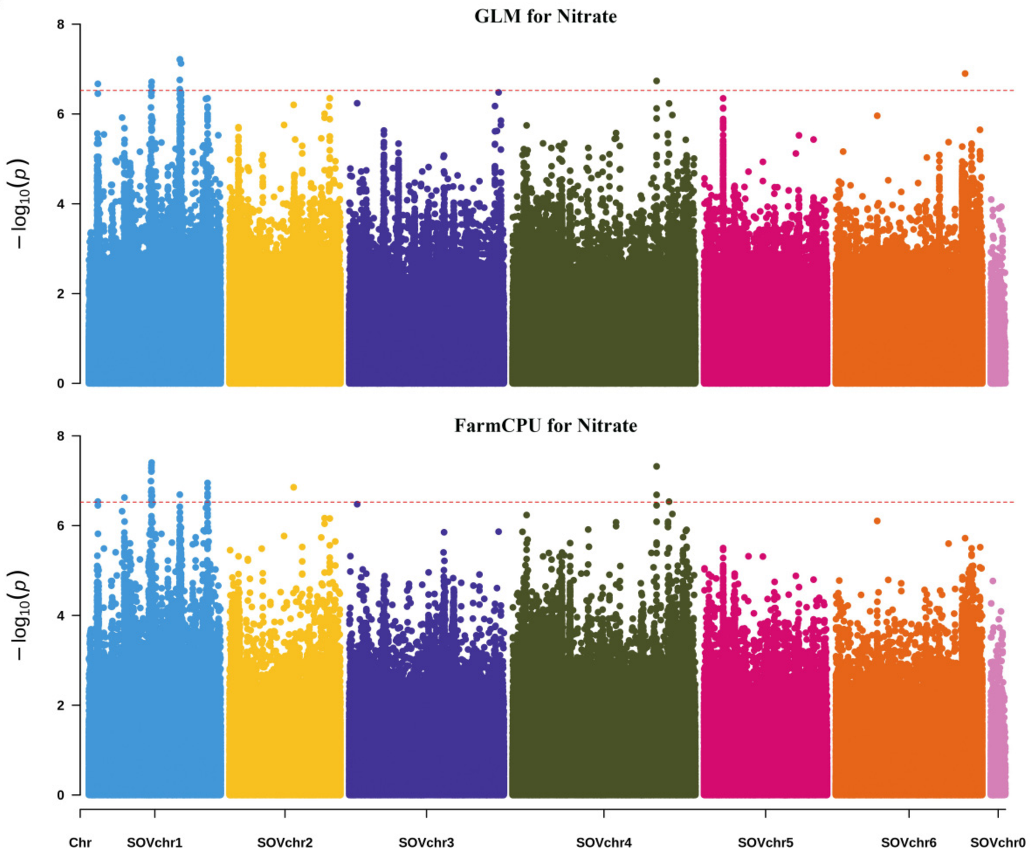
GLM for Nitrate
FarmCPU for Nitrate
$-\log_{10}(p)$
8
6
4
2
0
Chr
SOVchr1
SOVchr2
SOVchr3
SOVchr4
SOVchr5
SOVchr6
SOVchr0

d

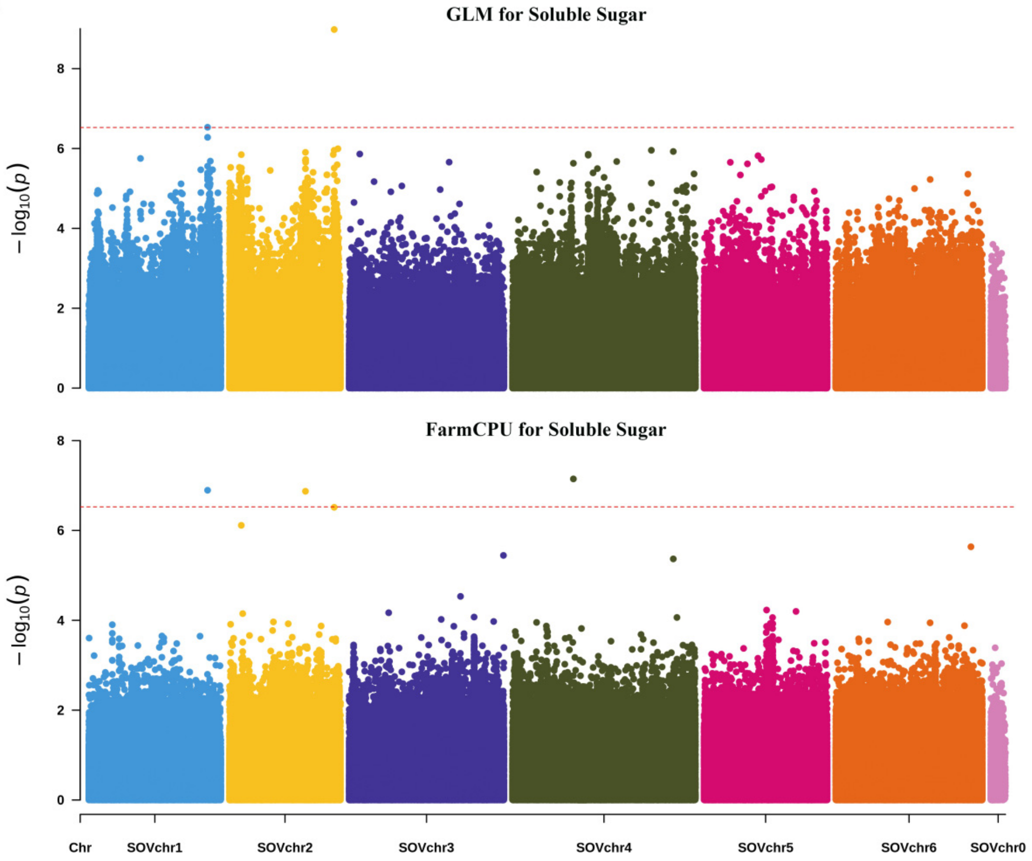

GLM for Soluble Sugar
FarmCPU for Soluble Sugar
$-\log_{10}(p)$
0
2
4
6
8
Chr
SOVchr1
SOVchr2
SOVchr3
SOVchr4
SOVchr5
SOVchr6
SOVchr0

e

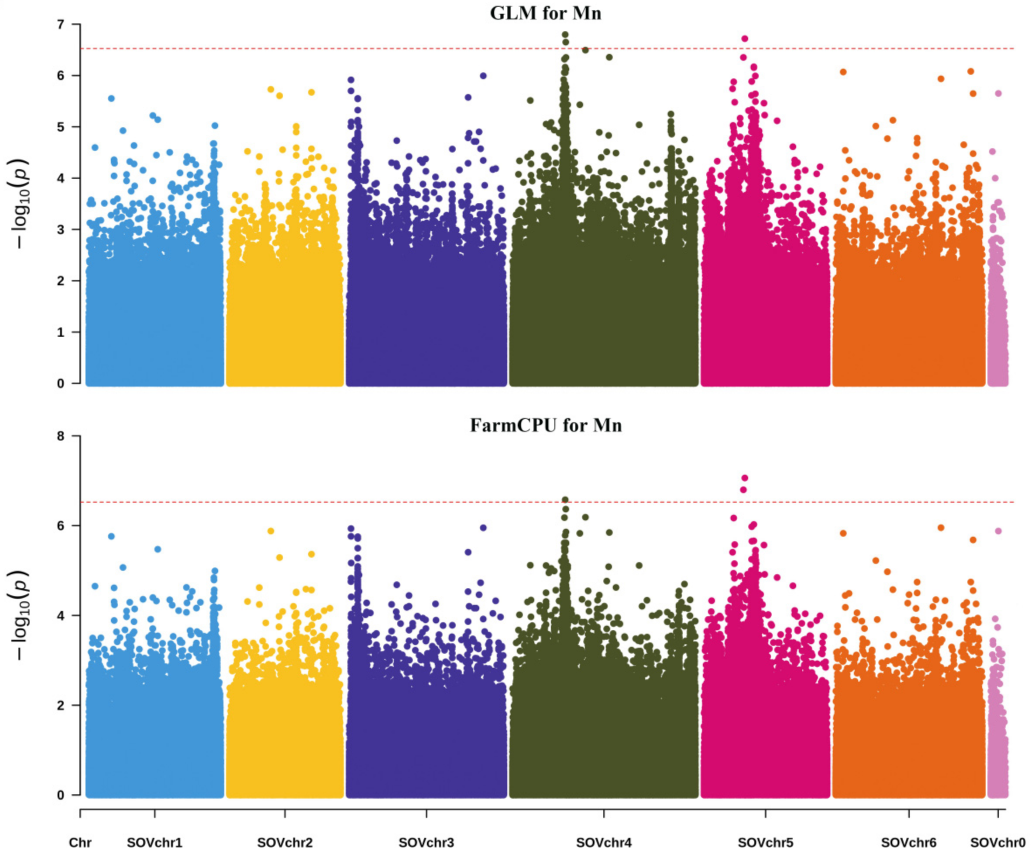
GLM for Mn
FarmCPU for Mn
$-\log_{10}(p)$
Chr
SOVchr1
SOVchr2
SOVchr3
SOVchr4
SOVchr5
SOVchr6
SOVchr0

f

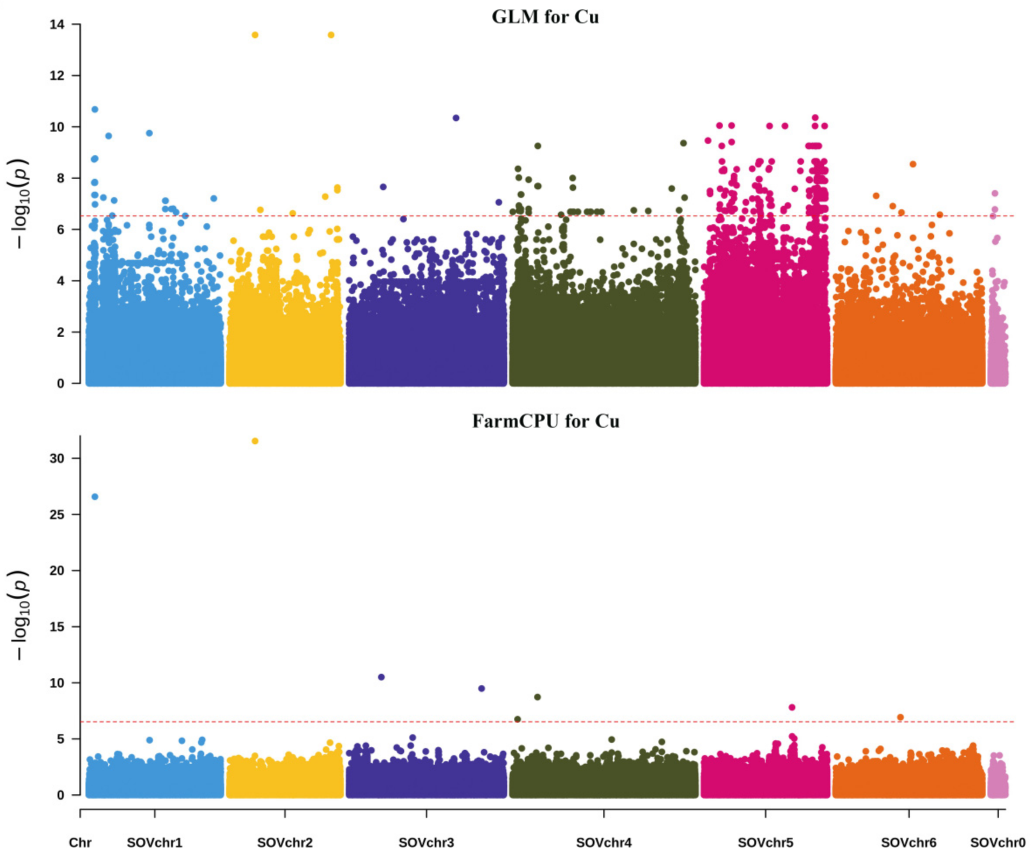

GLM for Cu
FarmCPU for Cu
$-\log_{10}(p)$
$-\log_{10}(p)$
Chr
SOVchr1
SOVchr2
SOVchr3
SOVchr4
SOVchr5
SOVchr6
SOVchr0

g

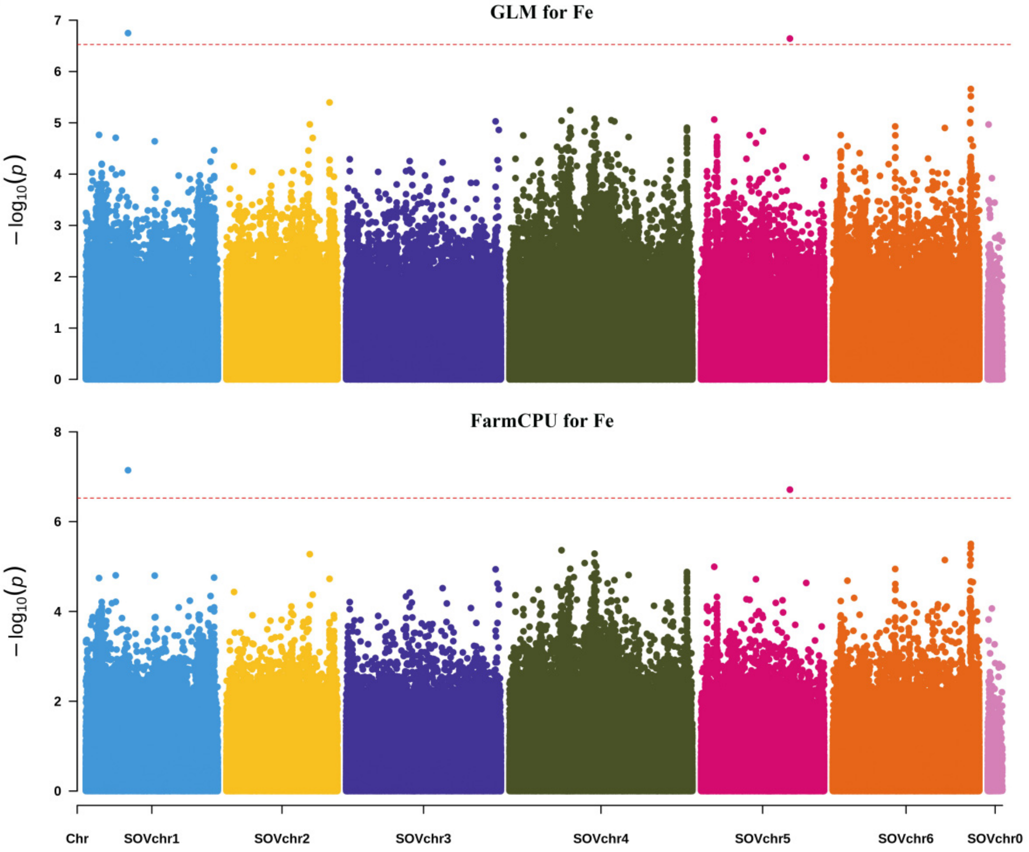

Figure S2: Manhattan plot for genome-wide association analysis (GWAS) of nutrients (a: chlorophyll a; b: chlorophyll b; c: nitrate; d: soluble sugar; e: Mn; f: Cu; and g: Fe) content in spinach using GLM and FarmCPU models. The horizontal and vertical axes represent the genomic position of the SNP and the association power for each SNP, respectively, with the trait expressed as $-\log_{10}(P)$. The dashed line shows the Bonferroni-corrected genome-wide threshold.
