# Supplementary figures and images for: A Genome-Wide Association Study Reveals the Genetic Mechanisms of Nutrient Accumulation in Spinach
Source: Genes (Basel). 2024 Jan 28;15(2):172. doi: 10.3390/genes15020172 (PMC10887921; doi:10.3390/genes15020172)

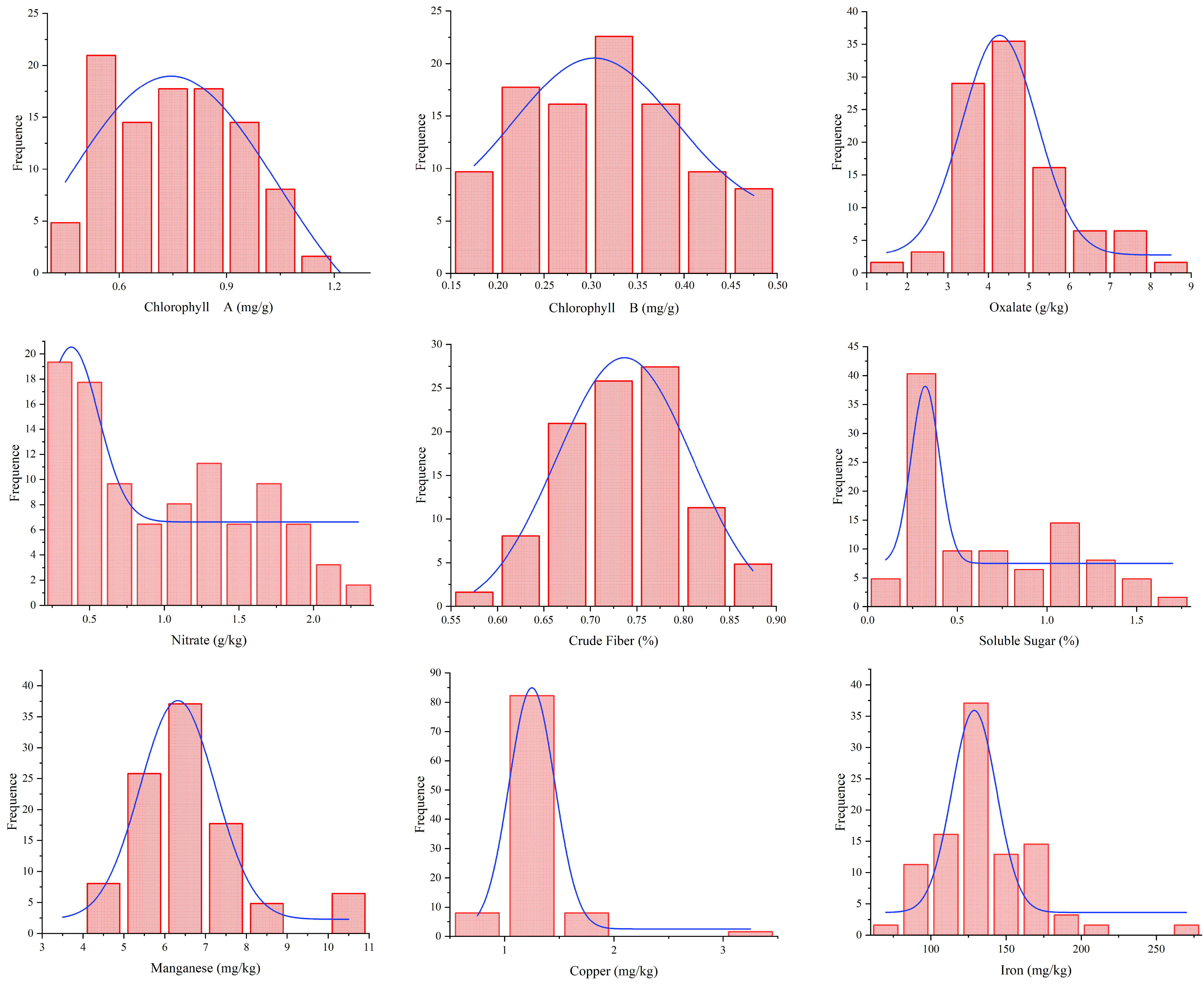

Supplement: Supplementary file 1 [file genes-15-00172-s001.zip › Figure S1.jpg]

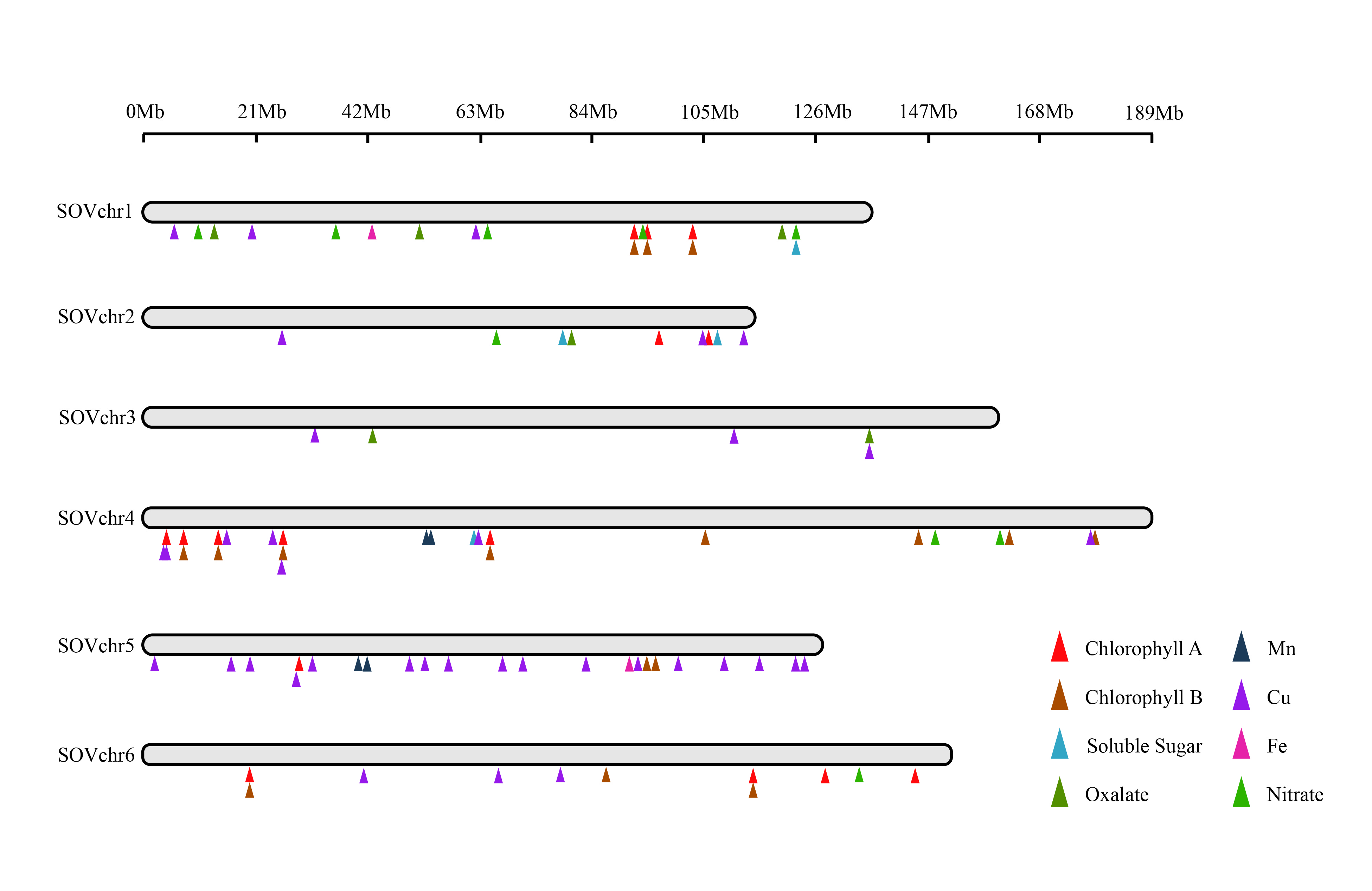

Supplement: Supplementary file 1 [file genes-15-00172-s001.zip › Figure S3.jpg]
